# Natural genetic variation shapes root system responses to phytohormones in Arabidopsis

**DOI:** 10.1111/tpj.14034

**Published:** 2018-09-10

**Authors:** Daniela Ristova, Marco Giovannetti, Kristina Metesch, Wolfgang Busch

**Affiliations:** ^1^ Gregor Mendel Institute (GMI) Austrian Academy of Sciences Vienna Biocenter (VBC) Dr. Bohr‐Gasse 3 Vienna 1030 Austria; ^2^ Salk Institute for Biological Studies Plant Molecular and Cellular Biology Laboratory, and Integrative Biology Laboratory 10010 N Torrey Pines Rd La Jolla CA 92037 USA

**Keywords:** natural variation, hormones, root growth, root system architecture, genome‐wide association study, development, *Arabidopsis thaliana*

## Abstract

Plants adjust their architecture by modulating organ growth. This ability is largely dependent on phytohormones. While responses to phytohormones have been studied extensively, it remains unclear to which extent and how these responses are modulated in non‐reference strains. Here, we assess variation of root traits upon treatment with auxin, cytokinin and abscisic acid (ABA) in 192 Arabidopsis accessions. We identify common response patterns, uncover the extent of their modulation by specific genotypes, and find that the Col‐0 reference accession is not a good representative of the species in this regard. We conduct genome‐wide association studies and identify 114 significant associations, most of them relating to ABA treatment. The numerous ABA candidate genes are not enriched for known ABA‐associated genes, indicating that we largely uncovered unknown players. Overall, our study provides a comprehensive view of the diversity of hormone responses in the *Arabidopsis thaliana* species, and shows that variation of genes that are yet mostly not associated with such a role to determine natural variation of the response to phytohormones.

## Significance Statement

Plant hormones are crucial for regulating root growth, but most studies were based on a single reference strain of Arabidopsis. Here, we study natural variation of root growth upon treatment with auxin, cytokinin and abscisic acid, and find that most strains respond very differently to hormones than the reference strain, and that this extensive natural variation is largely associated with genes that have not yet been implicated in playing a role in hormonal pathways.

## Introduction

To grow and survive, plants need access to sunlight, nutrients and water resources that are not evenly distributed in the environment. As sessile organisms, plants adjust their architecture according to the distribution of resources by modulating organ growth and development. Consequently, plant architecture is of major adaptive relevance (Ackerly *et al*., 2000). Directed plant growth responses, as well as the resulting plant architecture, are largely dependent on phytohormones, systemic signals that are interpreted in a cellular context (Malamy, 2005). Work related to the phytohormone auxin in a small number of *Arabidopsis thaliana* natural strains (accessions) has shown that this pathway is subject to extensive natural variation at the transcriptional level (Delker *et al*., 2010). However, it is unclear how this relates to plant architecture phenotypes, how the numerous other phytohormone pathways are affected by natural variation, and what the genetic bases for these alterations are. Moreover, it is not known whether the large number of phenotypic and associated molecular responses to phytohormones described in the reference accession (Col‐0) are representative for *A*. *thaliana* as a species.

The root is an excellent system for studying the dependence of plant architecture on phytohormone pathways. Not only is it technically feasible to perform hormonal perturbations on a large number of roots, but root traits are also of high adaptive relevance as the root system represents the backbone for plant growth and productivity. It anchors the plant to the soil, uptakes water and nutrients, and interacts with soil microorganisms (Lynch, 1995; Den Herder *et al*., 2010). There are three key developmental processes that determine the most important properties of the spatial distribution of roots, known as root system architecture (RSA): (i) the rate of proliferation and differentiation, which are the two main processes shaping how quickly roots grow; (ii) root growth direction, which determines in which direction the root system is expanding; (iii) the formation of lateral roots, which determine the lateral extensiveness of the root system. All of these processes are under the control of multiple phytohormone pathways (for an overview, see Satbhai *et al*., 2015). These hormonal pathways play a key role in adjusting RSA in response to environmental conditions, including availability of the three main macronutrients: nitrogen (Krouk *et al*., 2010; Vidal *et al*., 2010; Ruffel *et al*., 2011; Gifford *et al*., 2013; Rosas *et al*., 2013) phosphate (Lopez‐Bucio *et al*., 2005; Nacry *et al*., 2005; Perez‐Torres *et al*., 2008; Singh *et al*., 2014) and potassium (Vicente‐Agullo *et al*., 2004), as well as stresses such as salt (Zhao *et al*., 2011; Ding *et al*., 2015; Kumar and Verslues, 2015; Liu *et al*., 2015), drought (Kang *et al*., 2002; Du *et al*., 2012; Hong *et al*., 2013) and exposure to excess metals (Sun *et al*., 2010; Hu *et al*., 2013; Yuan *et al*., 2013). Despite the prominent role of phytohormones to determine root growth plasticity that is present in natural accessions in response to nutrient signals (Chevalier *et al*., 2003; Gifford *et al*., 2013; Kellermeier *et al*., 2013; Rosas *et al*., 2013), it is not known to which extent and how natural genetic variation affects the RSA response to different hormones.

Here, we assess natural variation of RSA traits upon treatment with auxin (IAA), cytokinin (CK) and abscisic acid (ABA), and generate and analyze a comprehensive atlas of these responses in a large set of accessions covering most of the genetic diversity in *A. thaliana*. We identify common response patterns and linked RSA traits that are controlled by specific phytohormone pathways, and uncover the extent of their modulation by specific genotypes. Using expression analysis, we show that the expression of key genes for multiple hormone pathways is frequently altered in accessions with contrasting RSA responses to hormones. We further show that known regulators of ABA signaling are not overrepresented in the set of genes associated with natural variation of root growth of seedlings treated with ABA, indicating that these genes are possible new players in hormone response pathways.

## Results

### The responses of root traits to exogenous application of hormones are subject to extensive natural variation

**Figure 1:**
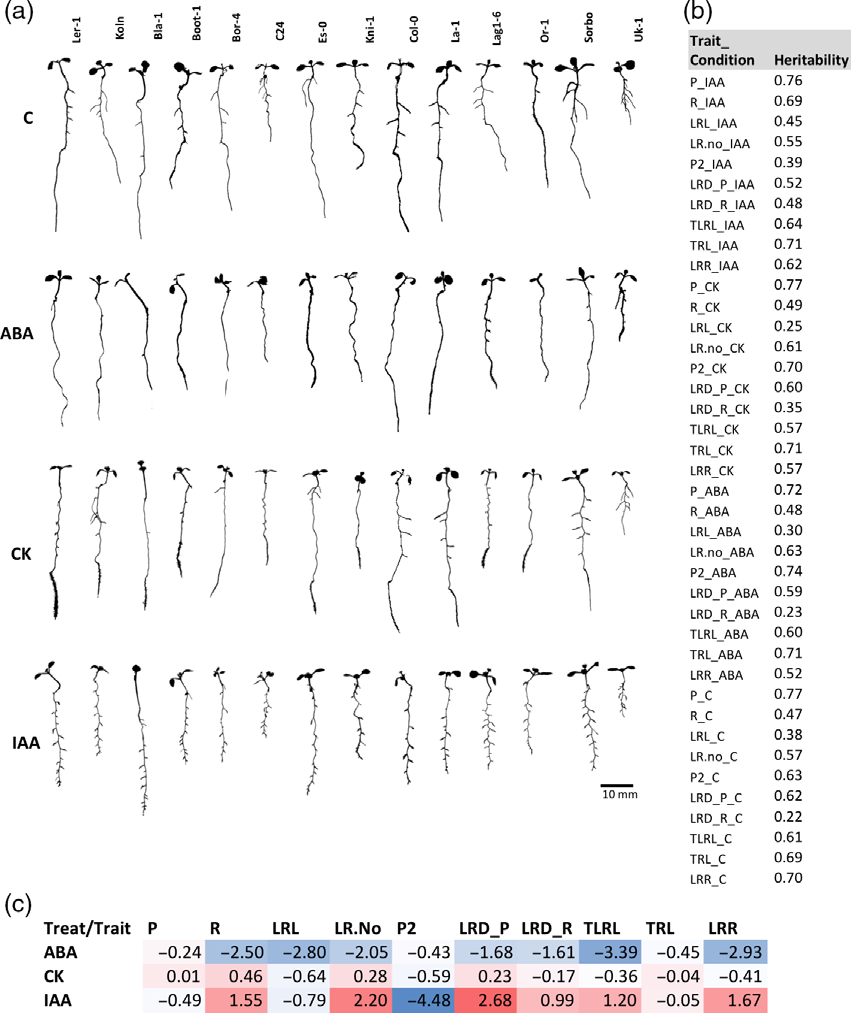
Representative root phenotypes of 14 Arabidopsis accessions on three hormone treatments and control.(a) One‐hundred and ninety‐two Arabidopsis accessions were grown on control conditions for 7 days followed by transfer to media supplemented with auxin (IAA), cytokinin (CK) and abscisic acid (ABA), or no hormone (C, control). Plates were scanned on day 10, and root traits were quantified and segmented using FIJI. Here, we show representative segmented seedlings of 14 accessions.(b) Broad sense heritability of all quantified root traits in all conditions.(c) Heatmap of (log_2_) fold‐change for each trait and hormone treatment compared with control treatment across all 192 accessions and four conditions. Ten root traits were quantified: primary root length (P), growth of P after the transfer (P2), branching zone (R), average lateral root length (LRL), lateral root numbers (LR.No), total lateral root length (TLRL), total root length (TRL), root density in P (LRD_P), root density in R (LRD_R), length ratio (LRR) (Figure S2b,c).

**Figure 2:**
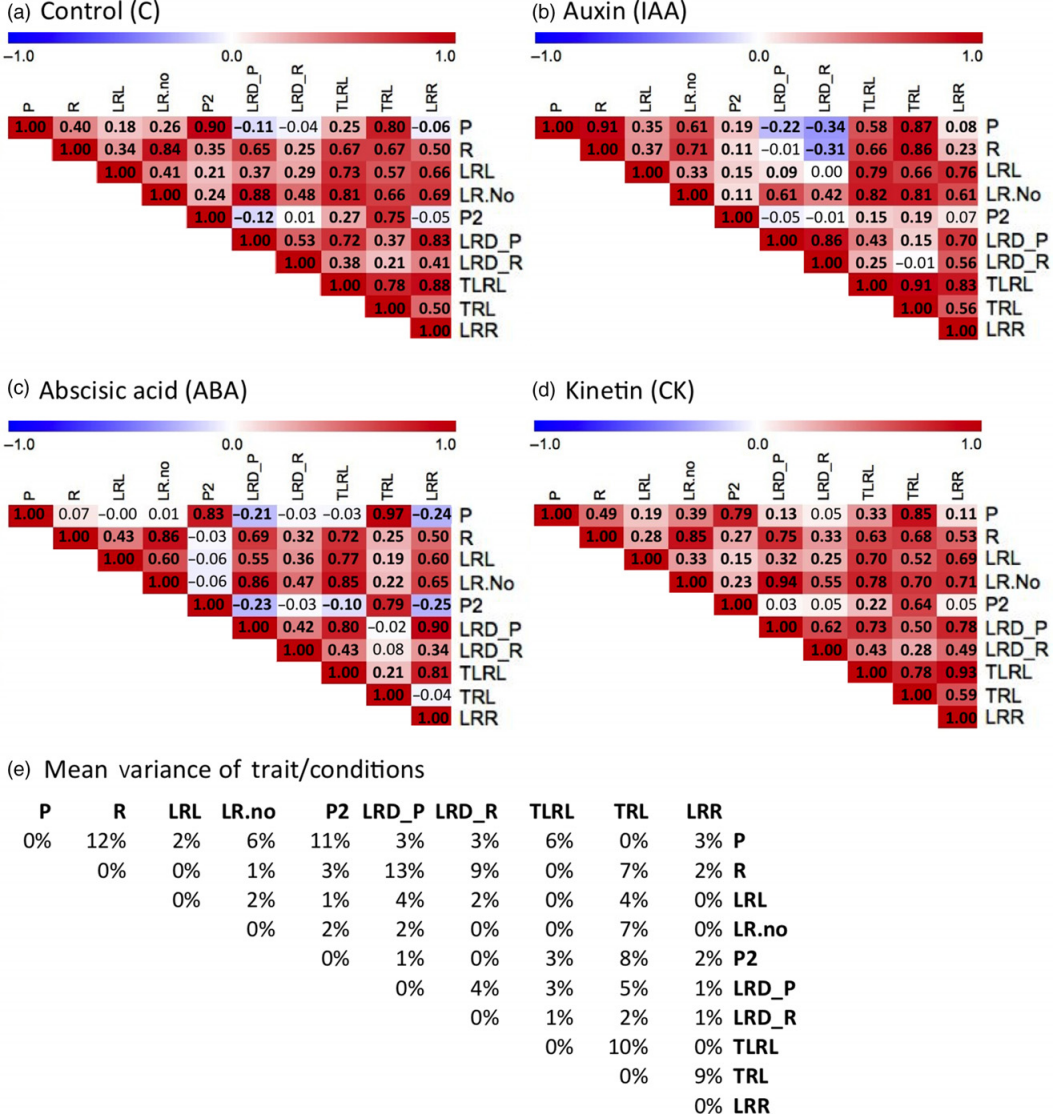
Patterns of root trait correlations.(a–d) Heatmaps of pairwise correlations (Pearson product‐moment correlation coefficient) of 10 root traits in 192 accessions upon transfer on control (C; a), auxin (IAA; b), abscisic acid (ABA; c) and cytokinin (CK; d) mediums. Color gradient indicates negative (blue) or positive (red) correlation coefficients, while bold numbers represent significant correlations (< 0.0001).(e) Mean variance of all trait correlations as a percentage. Traits: primary root length (P), growth of P after the transfer (P2), branching zone of P (R), average lateral root length (LRL) and lateral root numbers (LR.no). Other traits are calculated by formulas: total LRL, TLRL (LR.L*LR.No), total root length, TRL (TLRL + P), density of P, LRD_P (LR.No/P), density of R, LRD_R (LR.No/R), and length ratio, LRR (TLRL/P).

In order to study and catalog natural variation of phenotypic responses to phytohormones, we set out to devise a reproducible assay that was suitable for being conducted at a large scale. For this, we aimed to reduce batch effects between different accessions by transferring all seedlings undergoing one hormonal treatment at the same day. Therefore, we had to work with a relatively low number of seedlings. To reduce heterogeneity among these seedlings, we grew 10 seedlings on one‐fifth‐strength Murashige and Skoog (MS) medium on vertical plates for 7 days, and then transferred a subset of five seedlings that were closest to the mean of the 10 individuals onto plates with the treatment condition. One potential concern arising due to the low number of replicates was that the position of a seedling on a plate could impact root traits. We therefore tested the effect of placement in the upper versus the lower row on the plate with five seedlings in two independent experiments (Figure S8). As we did not find a significant difference between the rows within a plate for the tested accessions and hormone concentrations, we concluded that our setup was suited to determine trait variation among the different genotypes. Using this approach, we then grew a diverse panel of 192 Arabidopsis accessions covering most of the Arabidopsis genetic diversity (Figure S1; Table S1). After 7 days, we carefully transferred individual seedlings to different plates supplemented with IAA, CK, ABA or no hormone (C) (Figures 1 and S2a). Plates were scanned directly after the transfer and then again 3 days later. These images were used to quantify 10 root traits (Figure S2b,c). Overall, we quantified five seedlings of 192 accessions in four different conditions (three hormone treatments plus control), totaling 3840 seedlings and 38 400 root measurements (see Experimental procedures). There was a very broad spectrum of responses of root growth and development upon perturbation of the endogenous hormone pathways (Figures 1 and S3). Importantly, these responses were clearly dependent on the genotype, as the broad‐sense heritability of the traits was medium to high depending on the trait with an average of 57% for all traits and conditions, ranging from 22% for lateral root density in the branching zone under control conditions to 77% for the primary root length on CK treatment (Figure 1b). In our treatment conditions, we observed strong effects of IAA and ABA on most of the traits, while the impact of CK was rather subtle (Figure S3). IAA had a strong negative effect on root growth rate (P2), and strong positive effects on lateral root‐related traits (branching zone, R; lateral root number, LR.No; root density, LRD_P; total lateral root length, TLRL; and length ratio, LRR). ABA had strong negative effects on lateral root‐related traits (R, LRL, LR.No, LRD_P, LRD_R, TLRL and LRR), the opposite effect to IAA (Figure 2c). The strongest effect of CK was a negative effect on root growth (primary root growth rate, P2, and lateral root number, LRL); although we did not explicitly quantify it, we also observed a highly increased stimulation of root hair growth upon CK treatment in most of the accessions, especially on the elongated primary root (Figure 1a). Despite being quite genetically diverse, most accessions followed these general response patterns, with only some accessions showing strong deviations with regard to specific hormonal perturbations or traits. The reference accession, Col‐0, on which most of the previous studies of hormonal effects had been performed, is among the accessions whose root growth responses are frequently quite different from the bulk of Arabidopsis accessions. On average, it is in the upper quartile of the accession distribution and belonged to the 1% most extreme phenotypes for some traits in some conditions (Figure S3; Table S2). Col‐0 is therefore not a good representative of the diverse panel of accessions that we have investigated.

In conclusion, we have generated comprehensive atlas of root responses to perturbation of hormonal pathways. Our data show that while there are clear trends in phenotypic responses to perturbation of specific hormonal pathways, there is significant natural variation in these responses. Overall, this suggests that hormonal signaling pathways within the *A. thaliana* species are largely acting on the same traits, but that the extent of the hormonal control of specific traits is genotype dependent. We therefore conclude that hormonal pathways are subject to natural genetic variation.

### Distinct hormonal pathways dominate distinct traits

The degree of correlation between different traits in diverse genotypes is highly relevant for inferring the genetic architecture of these traits; a strong correlation suggests a common genetic, and possibly molecular, basis, underlying the regulation of the respective traits. Our comprehensive atlas of RSA traits of a large number of Arabidopsis genotypes upon perturbation of phytohormone pathways allowed us to ask which of the hormonally regulated RSA traits was linked and to what extent. We used our trait data from the 192 accessions and calculated pairwise Pearson's correlation coefficients of all traits for each condition (Figure 2a–d). To determine whether the extent of trait correlation was similar in all conditions, or whether perturbations of phytohormone pathways affected a particular trait correlation, we also calculated the variance of each trait correlation between all conditions. We reasoned that if this variance was high, the observed trait correlation would be strongly dependent on a subset of phytohormone pathways (Figure 2e). The highest variation of correlations between independent traits was observed for the length of branching zone (R) and the lateral root density (LRD_P) of the primary root (*σ* = 0.13). While these traits are highly correlated in control (0.65), CK (0.75) and ABA (0.69) conditions, this correlation reverses upon IAA treatment (−0.01). This strong effect can be traced back to the impact of IAA on root growth rate and its impact on the branching zone, which is a key for the second and third ranked correlation variances, respectively. Correlation analysis strongly suggests that the regulation of root growth rate is strongly dominated by the IAA pathway, as previously demonstrated by multiple studies (Evans *et al*., 1994; Coenen and Lomax, 1997; Tian and Reed, 1999; Friml, 2003; Swarup and Bennett, 2003; Robert and Friml, 2009; De Smet *et al*., 2012; Pacifici *et al*., 2015). Similarly, in our conditions, we found that ABA generally represses the growth of lateral roots, a finding that is also consistent with the literature (De Smet *et al*., 2003).

Overall, these examples not only demonstrate the significance of specific hormone pathways for specific traits, but also support the view that RSA traits such as branching (R) and primary root growth rate (P2) can be regulated independently of each other, dependent on the condition, and over a large number of genetic backgrounds, a phenomenon previously shown for the impact of different nitrogen environments on root traits (Gifford *et al*., 2013).

### Genotypic variation determines phenotypic responses to hormones

**Figure 3:**
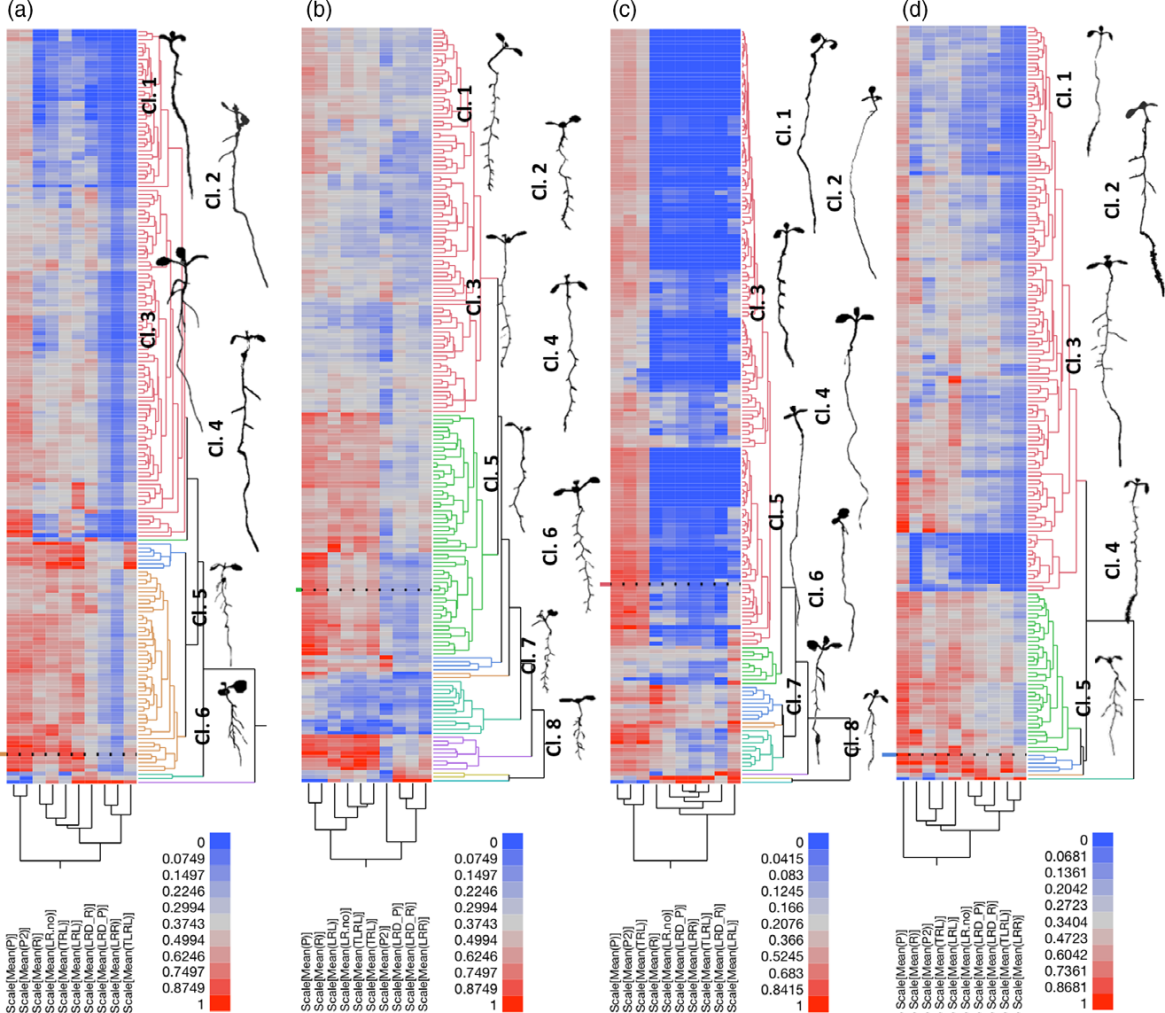
Hierarchical clustering (HC) of Arabidopsis accessions by condition.HC was performed on scaled means of 10 root traits (columns in heatmap) of 192 accessions (rows in heatmap), in each of the conditions [control (C), abscisic acid (ABA), cytokinin (CK) and auxin (IAA)]. On the right side of each dendrogram, we show one representative segmented plant of one accession from each cluster (Table S3). Distinct clusters are marked in different colors and represent groups of accessions with similar root architectures under the respective conditions.(a) HC of 192 accessions on C (control, no hormone added). (b) HC of 192 accessions on IAA. (c) HC of 190 accessions on ABA. (d). HC of 192 accessions on cytokinin (CK). Below each dendrogram, the corresponding heatmap legend is shown, indicating the value distribution for each condition. In each dendrogram, the reference accession (Col‐0) is marked with a dashed line. Cluster numbers and additional info are in Table S3.

**Figure 4:**
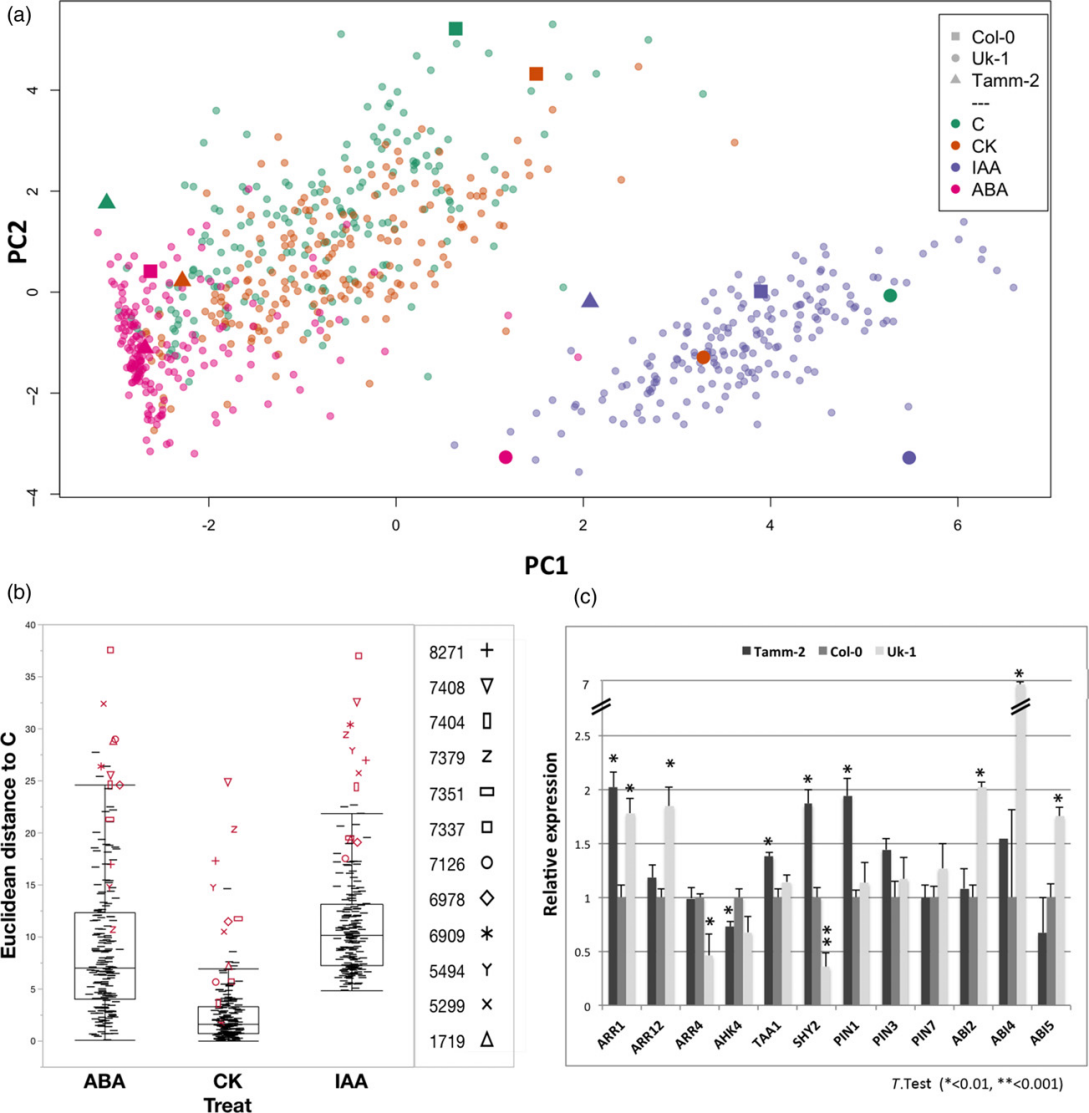
Multivariate analysis of 192 Arabidopsis accessions on four conditions and gene expression analysis from extreme accessions.Mean values for 10 root traits across 192 Arabidopsis accessions and four conditions [auxin (IAA), cytokinin (CK), abscisic acid (ABA), and no hormone (C)] were used to perform a principal component analysis (PCA).(a) Biplots of the 10 quantified root traits and 192 accessions under four conditions are shown for the first two principal components (PCs). Each accession in each condition is represented by a dot, and each condition has a different color. Highlighted are three contrasting accessions using different symbol shapes: Tamm‐2 is the accession with the most negative value for PC1 under control condition; Col‐0 is the reference accession; Uk‐1 is the accession with the most positive value for the first PC under control condition.(b) Response by hormone treatment relative to control, quantified by the Euclidean distance by the first two principal components (PCs), in 192 accessions. The 12 most deviating accessions are depicted by different markers and red color.(c) Relative expression of 12 genes involved in IAA, CK and ABA hormonal signaling pathways in extreme accessions quantified by PC1: accession Tamm‐2 (negative extreme in PC1), accession Uk‐1 (positive extreme in PC1), and the reference accession Col‐0. Segmented seedling phenotypes of the three accessions are shown at the top, including the PC1 value, and the position of each accession in control conditions is circled in (a). A Student's *t*‐test was performed to determine the significance of gene expression changes in the extreme accessions compared with the reference accession. Mean with SEM is shown. Data from three–four biological and two technical replicates. *P < 0.01.

While our correlation analysis revealed common trends over a large number of genotypes, our atlas of root responses to perturbation of hormonal pathways also allowed us to study which groups of genotypes modulate the responses to perturbations of hormone pathways. When conducting two‐way anova analysis on the data set, we found that there was a significant genotype treatment interaction for all traits and in all treatments (G*T Interaction *P* < 0.0001). To explore the genotype by environment interaction with regard to RSA, we performed a hierarchical clustering of the 10 traits separated by condition. Different clusters contain groups of accessions with genotypes that generate a similar RSA under the respective conditions (Figure 3;Table S3). We observed that perturbation of specific hormone pathways shifts RSA toward a distinct morphology (e.g. IAA causes roots to become shorter and more branched). However, the genotype determines the degree to which RSA is shifted in this direction. Our analysis partitions the phenotypic space that we explored using the systematic perturbation of hormone pathways, and illustrates the profound impact of the genotype in determining how root development responds to hormones. However, while this analysis is highly useful for identifying classes of accessions and visually illustrates the effects of hormones and how these effects differ, it was based on capturing RSA using individual traits, some of which are dependent on each other and some of them not. Moreover, hierarchical clustering gives insight into classes, rather than continuous similarities. To gain a more integrative insight into the action of hormones, we conducted a principal component analysis (PCA) on all our traits for all conditions (10 root traits across 192 accessions and four treatments). About 99% of RSA variation (given by our 10 traits) could be captured by five principal components (PCs), while nine PCs are needed to explain the complete variation (Table S4). The first PC captures 62% of the total variation. Several traits contribute to it, including root number (LR.No), lateral root density (LRD_P and LRD_R), total lateral root length (TLRL), and length ratio (LRR). The second PC accounts for 26% of the variation, and represents mainly primary root length (P) and total root length (TRL; Table S4). Overall, IAA treatment, and to a lesser extent ABA treatment, results in a clear separation from the other three conditions when plotting the most informative PCs, PC1 and PC2, while CK and C are largely overlapping (Figure 4a). To identify accessions with genotypes that alter from the norm of how RSA responds to hormone pathway perturbation, we calculated the Euclidian distance (Ed) in the RSA space defined by PC1 and PC2 for each accession from each hormone treated to the control treated sample (Table S5; Experimental procedures). This distance indicates the effect of the treatment in relation to its RSA under control conditions and thereby is corrected for developmental differences of the accessions (Figure 4b). For each accession, we then calculated the average Ed for all conditions (Table S5). This average represents a measure of how profoundly a genotype differs from the norm in altering its RSA in response to all hormonal perturbations that we had performed. The most average accession was Benk‐1. Twelve accessions (Es‐0, Ty‐0, Wc‐1, Wa‐1, KBS‐Mac‐16, UKNW06‐259, Col‐0, Uk‐2, Bu‐0, UKSE06‐565, Si‐0 and Wt‐3) were more than two standard deviations from the mean of all accessions in this regard (Figure 4b; Table S5), indicating that they show significant alterations of their hormonal response from the average *A. thaliana* accession. Again, the Col‐0 reference accessions were among these accessions, once more highlighting that the Col‐0 accession does not represent the species as a whole with regard to growth responses to hormone treatments. Taken together, these data demonstrate that hormone perturbations generally lead to specific patterns of developmental responses that shift RSA from one configuration to another. However, these response patterns can be significantly altered by genotypic variation. This genotypic variation must therefore be in the pathways that perceive or respond to phytohormone cues. Overall, this demonstrates that natural genetic variation in phytohormone pathways can be a major contributor in shaping the Arabidopsis root system.

### Genetic variation leads to changes in the molecular regulation of hormone pathways

To test whether the phenotypic differences we observed are reflected at the molecular level, we measured the expression of genes related to hormone signaling in accessions that represented different PC1 values (Uk‐1: high PC1; Tamm‐2: low PC1; Col‐0: intermediate PC1; Figure 4a). We included eight genes of the *SHY2* (*SHORT HYPOCOTYL 2*) network that are involved in IAA, CK and ABA signaling, and four additional genes involved in IAA biosynthesis (*TAA1, TRYPTOPHAN AMINOTRANSFERASE OF ARABIDOPSIS 1*; Stepanova *et al*., 2008), ABA signaling (*ABA2, ABA DEFICIENT 2*; Leung *et al*., 1997) and CK signaling (*ARR4, RESPONSE REGULATOR 4; AHK4, ARABIDOPSIS HISTIDINE KINASE 4*; Yamada *et al*., 2001; Salome *et al*., 2006). We found that eight of the 12 genes were expressed at different levels in these accessions, notably expression of *SHY2*, a key regulator for IAA and CK pathways in the root, showed an inverse relation to the PC1 values (low in Uk‐1, high in Tamm‐2; Figure 4c). Thus, the steady‐state expression level of important components of hormone pathways can be vastly different in different accessions. To corroborate this finding, we also made use of a public data set of root transcriptome data of seven accessions (Delker *et al*., 2010). Under control conditions, there was also significant variation in expression of these key genes, providing further evidence that the expression levels of genes in hormonal pathways are highly dependent on the genotype (Figure S4). Overall, this demonstrates that there can be a wide variety of transcriptional states in hormonal pathways relevant to RSA in different genotypes.

### Genes associated with root growth under hormone treatments are not enriched for *bona fide* candidate genes

Our analysis of root responses to hormone pathway perturbation clearly showed that these responses are under genetic control. To identify genetic variants that were associated with the different hormone treatments, we performed genome‐wide association (GWA) mapping on all traits in all conditions (40 traits; Table S9) using a mixed model algorithm (Yu *et al*., 2006; Kang *et al*., 2008) that was previously shown to correct for population structure confounding (Seren *et al*., 2012), and using SNP data from the 250K SNP chip (Atwell *et al*., 2010; Brachi *et al*., 2010; Horton *et al*., 2012). We used a Benjamini–Hochberg threshold (5% FDR) to select significant associations. In total, we identified 114 significant associations, corresponding to 441 genes within a 20‐kb window of the associated SNPs (Figure S5; Tables 1 and S6). We found associations for each treatment, with ABA perturbation yielding the most at 98; CK had five significant associations, and IAA only one. GWA studies (GWAS) for the control condition without hormone treatment added nine associations (Tables 1 and S6). We observed only three cases where GWAS identified the same SNP associated with different treatment conditions and the same trait (Figure S5).

**Table 1:** Summary information of significantly associated regions obtained by GWA mapping

Trait	Treat	Ch #	SNP position	GWA *P*‐value	Benjamini– Hochberg (neg[log]Pval)
Length ratio (LRR)	C	3, 3, 5, 3	18730847, 6127399, 20263253, 9654227,	3.82E‐09, 7.70E‐08, 1.02E‐07, 3.11E‐07	5.65E+00
		1, 5, 5, 1	20162056, 25855852, 6909572, 18708566	5.02E‐07, 1.03E‐06, 1.25E‐06, 2.09E‐06	
Density in R (LRD_R)	C	1	16104248	6.63E‐08	6.31E+00
Average lateral root length (LRL)	IAA	3	23029970	1.89E‐07	6.31E+00
Length ratio (LRR)	CK	4, 3, 3, 3,	5502826, 6127399, 6128629, 1518618,	1.79E‐08, 7.70E‐08, 4.69E‐07, 5.05E‐07	5.94E+00
		5	17211166	5.05E‐07	
Lateral root number (LR.no)	ABA	1, 5	3464235, 21621187	1.88E‐07, 4.64E‐07	6.05E+00
Density in P (LRD_P)	ABA	3, 3, 3, 3,	18730847, 6432520, 10035920, 11140451,	2.00E‐07, 2.77E‐07, 3.85E‐07, 5.23E‐07	5.96E+00
		3	6432784	9.74E‐07	
Density in R (LRD_R)	ABA	1, 5, 3, 1,	3476564, 21621187, 21283684, 26910129,	1.23E‐07, 1.65E‐07, 2.44E‐07, 3.43E‐07	5.37E+00
		3, 1, 5, 5,	10995702, 3474640, 15450107, 21524212	3.53E‐07, 7.12E‐07, 7.27E‐07, 8.41E‐07	
		3, 1, 1, 4,	21258706, 2318710, 3476243, 13282571,	8.83E‐07, 1.01E‐06, 1.09E‐06, 1.12E‐06	
		1, 1, 4, 5,	5838544, 22271511, 13296141, 9498624,	1.20E‐06, 1.87E‐06, 2.01E‐06, 2.17E‐06	
		5, 1, 1, 4,	9067032, 5821121, 11953256, 9368143,	2.17E‐06, 2.41E‐06, 2.45E‐06, 2.50E‐06	
		4, 2, 1, 5,	3926453, 8276689, 17562670, 22270694,	3.71E‐06, 3.80E‐06, 3.98E‐06, 4.03E‐06	
		1	3473536	4.07E‐06	
Length ratio (LRR)	ABA	3, 5, 3, 3,	10035920, 14857304, 11140451, 6432520,	6.21E‐10, 3.51E‐09, 1.08E‐08, 1.17E‐08	5.47E+00
		3, 3, 3, 3,	18730847, 18368482, 6432784, 9984934,	1.39E‐08, 1.61E‐08, 3.02E‐08, 5.82E‐08	
		1, 1, 4, 1,	27053227, 6126365, 16527439, 27026361,	7.51E‐08, 7.60E‐08, 7.70E‐08, 1.66E‐07	
		3, 5, 2, 3,	3036751, 21515990, 16222279, 9999583,	1.86E‐07, 3.24E‐07, 3.73E‐07, 4.54E‐07	
		1, 1, 4, 3,	16504751, 25156407, 15708104, 9802078	5.75E‐07, 6.83E‐07, 8.70E‐07, 9.05E‐07	
		3, 1, 3, 3,	9804462, 26583045, 6431861, 7945274,	9.05E‐07, 9.11E‐07, 1.04E‐06, 1.08E‐06	
		3, 4, 5, 2,	9791884, 12837993, 25847727, 610291,	1.17E‐06, 1.28E‐06, 1.33E‐06, 1.39E‐06	
		5, 3, 1, 1,	22270694, 9799941, 25162621, 10387202,	1.39E‐06, 1.48E‐06, 1.60E‐06, 2.27E‐06	
		5, 3, 3, 5,	8248681, 10054595, 11153455, 8243957	2.39E‐06, 2.39E‐06, 2.41E‐06, 2.43E‐06	
		3, 1, 1, 3,	15049139, 2439854, 2439731, 21618856	2.56E‐06, 2.82E‐06, 2.82E‐06, 3.29E‐06	
Total lateral root length (TLRL)	ABA	3, 1, 5, 1,	10035920, 3464235, 8509007, 3476564,	2.20E‐09, 8.78E‐09, 3.05E‐08, 3.98E‐08	5.51E+00
		5, 5, 5, 5,	8525033, 8512551, 8513042, 3454021,	4.00E‐08, 5.28E‐08, 7.41E‐08, 8.37E‐08	
		1, 3, 1, 1,	3474640, 11140451, 3476243, 22084145,	1.87E‐07, 2.05E‐07, 3.87E‐07, 4.90E‐07	
		1, 4, 5, 5,	22084211, 8096866, 14857304, 8524288,	4.90E‐07, 5.65E‐07, 6.84E‐07, 1.23E‐06	
		1, 3, 5, 5,	6126365, 6431861, 21520565, 21525637,	1.24E‐06, 1.24E‐06, 1.45E‐06, 1.45E‐06	
		3, 5, 2, 3,	6432784, 21524545, 13725657, 18730847,	1.47E‐06, 1.86E‐06, 2.13E‐06, 2.22E‐06	
		1, 5	3474489, 21527231	2.48E‐06, 2.90E‐06	

GWA mapping (BH threshold, 5% FDR) was conducted on 10 root traits among 192 Arabidopsis thaliana accessions and four conditions (Control, C; auxin, IAA; cytokinin, CK, and abscisic acid, ABA). GWA, genome‐wide association; SNP, single nucleotide polymorphism.

Despite the large number of significant associations for the ABA pathway, the genes close (20‐kb region around each significant SNP) to these associations did not contain obvious core ABA signaling‐related genes. To test whether this absence of *bona fide* ABA candidate genes was statistically significant, we identified all genes for which a role in the ABA pathway had been assigned based on reported experimental evidence or mutant phenotypes (Table S7). There was only one gene that overlapped between GWAS‐based candidate gene list (20 kb around each significant SNP) and the annotation‐based ABA *bona fide* candidate gene list. We then tested whether this overlap is expected by chance, we found that our observed overlap was at the lower limit (toward our GWAS list being depleted of *bona fide* candidate genes) of that expected by chance (Figure S6). This showed that *bona fide* candidate genes are not overrepresented in our list of GWAS candidates and indicate that, under our screening conditions, new, previously non‐identified genes were found.

## Discussion

### Natural variation of hormone signaling pathways and root architecture

In this study, we report a comprehensive atlas of RSA responses to hormonal perturbations in a large number of *A. thaliana* natural accessions. While in particular for traits that showed a heritability within the lower heritability range of our data (Figure 1b), the use of more seedlings replicates might have increased the accuracy of the trait value, many traits showed a high heritability and could thereby be accurately captured using our experimental setup. Moreover, robust reproducibility of root growth under our conditions is indicated by a comparison of Col‐0 data that were obtained during the GWAS screen and Col‐0 measurements under the same conditions acquired 27 months later and by different experimenters. While the length of the roots that were transferred (P) was different, the root length increase after treatment (P2) with either C or CK was statistically not different between both rounds of experiments (Figure S10). This indicates that despite differences in the experimenters and certain uncontrolled environmental variables related to the 27 months between the experiments, treatment effects are reproducible. Nevertheless, it should be emphasized that variation of hormone treatment concentrations and other experimental factors such as agar, nutrient levels and light, growth responses to hormone treatments most certainly will lead to changes in the observed responses. However, we do not expect that the broad relations between the accessions, as well as their major differences will change dramatically within a reasonable parameter range.

Overall, our results show general patterns of phenotypic responses to the perturbation of specific hormonal pathways, as well as significant natural variation in these responses across a set of accessions that captures a large fraction of the genetic variation in Arabidopsis (Figure S1). We show that specific hormonal pathways dominate a distinct subset of traits, as perturbations of specific hormone pathways can break correlations that exist between traits under control conditions (Figure 2e). In our experimental conditions, IAA perturbation caused the most dominant effect on RSA by strongly repressing root growth rate and stimulating lateral root growth. ABA treatment reduced primary root length, as well as lateral root numbers (Figure 1b), and diminished the correlation between these two traits, thereby demonstrating its negative role in the emergence of lateral roots. In contrast to the replication of these well‐described effects of IAA and ABA (Evans *et al*., 1994; De Smet *et al*., 2003), our CK treatment conditions did not lead to the previously observed strong inhibitory effects on primary and lateral root growth (Li *et al*., 2006; Laplaze *et al*., 2007). We did, however, observe the stimulatory effect of CKs on root hair growth, which had been described before (Werner *et al*., 2001). A possible reason for the observed CK response in our experimental conditions might be the specific CK chosen and the concentration of nutrients in the medium, as we used kinetin and one‐fifth‐strength MS medium. Because we quantified only visible lateral roots, another reason might be that in our experimental conditions the exposure time to CK was too short to observe the phenotypes described earlier, as CK application does not affect already initiated lateral roots (Li *et al*., 2006; Laplaze *et al*., 2007). Regardless of the specifics of our treatments, our atlas of root growth responses revealed the extent of variation in the responses to perturbation of hormone pathways. Our hierarchical clustering results identified groups of accessions sharing similar or diverse responses to a particular hormone perturbation (Figure 3). Importantly, these clusters can be very useful for choosing parents for quantitative trait loci mapping via linkage mapping approaches using recombinant inbred lines. While current mapping populations are based on only a small subset of accessions (Alonso‐Blanco and Koornneef, 2000; Koornneef *et al*., 2004), they have already been used to identify *BREVIS RADIX* (*BRX*), a gene that is responsible for the hormone‐related short‐root phenotype of the UK‐1 accession (Mouchel *et al*., 2004, 2006). Our results provide an opportunity for choosing previously unexplored accessions with diverse root responses to hormones as parents for creating new populations to identify alleles underlying hormone pathway‐dependent root developmental processes. PCA showed that hormones can shift RSA through the phenotypic space (Figure 4a). In our conditions, IAA treatment led to the most pronounced phenotypic effect on RSA compared with ABA and CK. Of course, we cannot exclude that other concentrations of hormones and their interplay with nutrient concentrations could lead to even more pronounced CK or ABA effects, but our findings corroborate the prominent role of IAA in root development. Moreover, the PCA analysis allowed us to systematically identify most deviating accessions that behave clearly differently than the bulk of Arabidopsis accessions with regard to the extent of the RSA response to hormone treatments (the general direction of the hormone response is not changed: e.g. IAA treatment still leads to decrease of primary root length and an increase in branching). One of these deviating accessions is the reference strain Col‐0 (Figures 3 and 4; Tables S2, S3, and S5), on which the vast majority of Arabidopsis studies are performed. This is not simply a peculiarity of our investigation; for example, in a study dissecting 107 diverse phenotypes (Atwell *et al*., 2010), Col‐0 is located in the 1% lower tail of the distribution of all accessions for 14% of the traits (Figure S9). While these findings do not affect the validity of the fundamental mechanisms for hormone responses that have mainly been discovered in Col‐0, they suggest that, at a certain level of detail, studies will uncover relations and mechanisms that are only found in specific genotypes within a species. On the other hand, using an outlier for generating populations for linkage‐based mapping approaches has clear benefits. We do not suggest Col‐0 to be among the most deviating accessions in all cases. In fact, for a small number of traits under some conditions in this study (Table S2), and for root traits in other studies, it has been described to be rather average (Rosas *et al*., 2013).

Importantly, accessions that are different with respect to their RSA response upon hormonal treatments also show deviations in the expression patterns of hormone‐related genes in control conditions (no hormone treatment). This shows that the varying responses we observed upon hormonal stimuli are associated with different transcriptional states of the hormone signaling networks. This conclusion is consistent with genome‐scale observations in seven accessions before and upon IAA treatment, in which vastly different transcriptome responses were detected (Delker *et al*., 2010). The gene expression differences of the hormone‐related genes for which we had quantified expression levels are only in the two‐ to fourfold range between different accessions. However, even such minor differences can indicate rather significant differences. For example, loss of function or overexpression of cytokinin response factor transcription factors have been shown to lead to two‐ to fourfold change effects on *PIN7*, while being associated with rather profound effects on root growth and development (Simaskova *et al*., 2015). Importantly, such different transcriptional states that presumably underlie altered responses to hormones can be caused quite indirectly, for example by alterations of the balance of another hormone pathway. This has been nicely illustrated by studies related to the *BRX* gene that is causal for a large proportion of the root phenotype of the UK‐1 accession. There, the lack of IAA response in the *brx* mutant results from a root‐specific deficiency in brassinosteroid production (Mouchel *et al*., 2006). Given the strong and widespread intraspecies variation that we have observed here, it is not surprising that distinct differences in the wiring and interaction of plant hormone pathway exist between species (Pacheco‐Villalobos *et al*., 2013).

Taken together, our results show that the root growth responses of Arabidopsis accessions upon the perturbation of hormone pathways display distinct patterns that depend on the hormone shifting RSA toward distinct states, and that the extent of this shift depends on genetic factors. This demonstrates that hormonal pathways are subject to natural genetic variation within a species, and it is tempting to speculate that this might contribute to adaptation to diverse soil environments.

### Genome‐wide association mapping and candidate genes

While phytohormone signaling pathways are fundamental mechanisms that are highly relevant for plant growth and development, only a few, small‐scale studies have addressed them in light of natural variation (West *et al*., 2007; Delker *et al*., 2010). Consequently, these studies were not able to map any of the causal variants underlying the remarkable natural variation of responses to hormones. Our GWAS analysis has detected more than 100 significant associations, with many good candidate genes among them. We have made all the data and GWAS publicly on the GWAS portal (https://gwas.gmi.oeaw.ac.at/), where these associations can be explored in depth and can serve as a basis for further investigation.

Using our data, we were able to search for common genetic variants that associate with natural variation of root growth under specific hormone treatments using GWAS. Using the large number of associations with ABA treatment, we found that genes already implicated in the ABA pathway were not enriched, and possibly even underrepresented, in our set of GWAS candidate genes (Figure S6). This poses the question if perhaps the traits that were measured were good indicators for ABA response. As we have largely observed responses that are similar to those described in previous studies (e.g. strong inhibition of lateral roots), this seems rather unlikely. Other possible explanations involve the presence of a notable number of false negatives (*bona fide* ABA genes that are causal but not detected by our GWAS analysis), or false positives (genes detected that are not causal), or a combination of both. By using a mixed model GWAS approach, which corrects for population structure and genetic relatedness, and a stringent threshold that was adjusted for multiple testing, we do not expect the false positive rate to be much higher than our false discovery rate threshold than 5% at the SNP level. However, population structure correction and statistical stringency can cause false negatives to occur. Moreover, because we included all genes in the 20‐kb window around a significant SNP in our analysis, we can expect that several non‐causal genes are in our list of candidates (thereby false positive candidates). However, even when taking only one gene per SNP into account, there is no significant enrichment of *bona fide* ABA genes. Nevertheless, we cannot fully exclude that part of an explanation for the lack of enrichment of *bona fide* ABA genes is due to false positives and false negatives. Future validation studies of the candidate genes identified here are needed to start to address this. One hypothesis to explain the lack of *bona fide* ABA genes without invoking a large number of unexpected false positives and false negatives is that mutations in *bona fide* ABA genes might be detrimental in the wild to some extent as known genes are often identified by their large effects that mutations cause. If that were true, *bona fide* ABA genes should display less natural variation than an average gene. Consistent with that idea, *bona fide* ABA genes display on average much less variation in their exons than the genes identified by GWAS in this study, as well as all genes (Figure S11).

## Experimental procedures

### Plant material and growth conditions

In total, 192 *A. thaliana* accessions were used from the Busch group collection at GMI, Vienna. The names and numbers of all accessions are listed in Table S1. Seeds were surface sterilized in a desiccator using 100 ml of household bleach and 3.5 ml of 37% HCl for 1 h. The seeds were stratified in water at 4°C in the dark for 3 days, and were sown on square Petri dishes containing 57 ml of growth medium. The control growth medium (before transfer) consisted of one‐fifth‐strength MS medium with MES buffer (Ducheva Biochemie, Haarlem, The Netherlands), 1% sucrose, 0.8% agar (Ducheva Biochemie), and adjusted to pH 5.7. Plates were dried for 45 min in a sterile laminar flow hood, before closing the cover and storing them at room temperature for 3 days before planting the seeds. In each plate, four accessions were plated, two in each row, with ten plant replicates per accession (Figure S2). Plates were placed vertically, and seeds were germinated under long‐day conditions (21°C, 16 h light/8 h dark cycle). Individual 7‐day‐old seedlings were carefully transferred to the same position onto plates containing control medium supplemented with ABA (1 μm), CK (1 μm) or IAA (0.5 μm) as indicated per experiment (Figure S2). Hormone concentrations have been chosen to be comparable with those used in previous studies to study changes in RSA as well as to identify genes implicated in hormone pathways controlling root responses (Nemhauser *et al*., 2006; Ristova *et al*., 2013, 2016). The particular screening conditions were chosen based on pilot experiments: before performing the experiment on all 192 accessions, we first tested the effect of different MS concentrations, as full‐strength MS medium was found to inhibit root growth (Dubrovsky *et al*., 2009) and was originally designed for tissue culture that includes very high concentrations of mineral nutrients (Dubrovsky and Forde, 2012). We indeed observed inhibition of root growth in the tested MS concentrations (Figure S7). Due to the larger number of accessions screened, we decided to use the lowest concentration tested, one‐fifth‐strength MS. Although development of lateral root primordia is previously reported at 4–5 days after germination, in our conditions and the accessions tested we did not observe visible (by eye) lateral roots before the 7th day. At day 10, the primary root of some of the accessions used in the study did outgrow the plate, thus we decided to quantify root traits after 3 days of treatment. These time points are comparable with previous study of RSA under hormone perturbations (Ristova *et al*., 2013, 2016).

### Phenotypic analysis

After 3 days of treatment, plates were scanned with CCD flatbed scanners (EPSON Perfection V600 Photo, Seiko Epson, Nagano, Japan), and images used to quantify root parameters with FIJI, by using the tool ‘segmented line’ (Schindelin *et al*., 2012; Figure S2) as described in Ristova and Busch (2017). In particular, we quantified: primary root length on day 10 (P), growth rate of P after treatment (P2), branching zone or the length of P between the first and last visible lateral root (R), average lateral root length (LRL), and visible lateral root number (LR.No). Length was measured in millimeters. Other traits were calculated: lateral root density of P, LRD_P (LR.No/P), lateral root density of R, LRD_R (LR.No/R), total lateral root length, TLRL (LR.No*LRL), total root length, TRL (TLRL + P), and length ratio, LRR (TLRL/P). For detailed protocol, see Ristova and Busch (2017).

### Genome‐wide association studies

We conducted GWA mapping on the mean trait values using a mixed model algorithm (Kang *et al*., 2008), which has been shown to correct for population structure confounding (Seren *et al*., 2012), and SNP data from the RegMap panel (Horton *et al*., 2012). SNPs with minor allele counts greater or equal to 10 were taken into account. The significance of SNP associations was determined at 5% FDR threshold computed by the Benjamini–Hochberg–Yekutieli method to correct for multiple testing (Benjamini and Yekutieli, 2001).

### Analyzing the overlap of abscisic acid *bona fide* genes and abscisic acid genome‐wide association study candidates

To determine a list of ABA *bona fide* candidate genes, we used the GO SLIM annotation of the TAIR10 release (ftp://ftp.arabidopsis.org/Ontologies/Gene_Ontology/ATH_GO_GOSLIM.txt; creation date: 4/9/15). We then filtered for gene ontology (GO) annotations that contained the key word ‘abscisic acid’ and were based on either IDA (‘inferred by direct assay’) or IMP (‘inferred by mutant phenotype’). The resulting list (Table S7) was subsequently used as the ABA *bona fide* candidate gene list. We then used the hypergeometric distribution to compute the *P*‐value for the observed overlap of the GWAS‐based ABA candidate gene list and the ABA *bona fide* candidate gene list. This was done in R (V3.1.2) using the command ‘dhyper(1,361,33602‐361,276)’, where 1 is the overlap of genes between the two lists, 361 is the number of genes in the genome that were found in the GWAS, 33602‐361 is the number of genes in the genome not found in the GWAS, and 276 is the number of genes in the genome that were *bona fide* candidate genes for ABA signaling. The same ‘dhyper’ analysis was conducted with the assumption of one gene per SNP by using 98 as the number of genes in the genome that were found in the GWAS.

### Analyzing polymorphism density

To determine the SNP density in exons, we used data from the 1001 genome project (Alonso‐Blanco *et al*., 2016) in which genes SNP summaries, as called by the intersection of SHORE and GATK pipeline, were provided at the population level (http://1001genomes.org/data/GMI-MPI/releases/v3.1/1001genomes_snpeff_v3.1/). To calculate the number of SNPs per exonic region of each gene, we divided for each gene the number of SNPs in all exons by the total exon length (bp).

### Statistical analysis of root traits

A biological replicate for phenotyping purposes is an independent seedling of the same genotype. For reverse transcription‐quantitative polymerase chain reaction (RT‐qPCR) experiments, a biological replicate consisted of bulked roots grown from the same plate and technical replicates were cDNA samples that were split‐up before RT‐qPCR and independently measured. RT‐qPCR was done with at least three–four biological and two technical replicates.

Hierarchical clustering was performed using the average linkage method, using scaled mean values of the 10 root traits across the accessions and hormone treatments. The smallest number of clusters for each condition was chosen based on the distance graph. PCA was performed on mean values of the 10 root traits across all accessions and conditions. The Ed for each treatment response was calculated for the first two PCs (two dimensions) for each accession between the control and the hormone treatments (IAA, CK and ABA) by the formula: Ed = SQRT ((PC1.C‐PC1.T)^2^ + (PC2.C‐PC2.T)^2^). The average Ed for all three hormone treatments was then calculated and transformed into a *Z*‐score to identify the most diverse accessions.

### Gene expression analysis (reverse transcription‐polymerase chain reaction)

Total RNA extraction was performed using a commercial RNA isolation kit (RNeasy Mini Kit Plus, QIAGEN, Hilden, Germany) using whole roots of 10‐day‐old seedlings, bulking 7–10 roots from the same accession/condition into one biological replicate. A qRT‐PCR reaction was prepared using 2 × SensiMix^TM^ SYBR & Fluorescein Kit (PEQLAB LLC, Wilmington, DE, USA), and PCR was conducted with a Roche Lightcycler^®^ 96 (Roche, Wien, Austria) instrument. Relative quantifications were performed for all the genes, and the β‐tubulin gene (AT5G62690) was used as an internal reference. Gene‐specific primers used are listed in Table S8. Previously described primer pairs were used for *ARR1* and *ARR12* (Moubayidin *et al*., 2010), *PIN3* and *PIN7* (Dello Ioio *et al*., 2008), and *TAA1* (Cui *et al*., 2013).

## Accession numbers

Phenotype data and GWAS analyses can be accessed via the GWAS portal (https://gwas.gmi.oeaw.ac.at/). Accession numbers for all 192 accessions of *A. thaliana* used in this study can be found in Table S1.

## Author contribution

DR: conception and design, contributed unpublished essential data or reagents, acquisition of data, analysis and interpretation of data, drafting or revising the article. MG: acquisition of data, analysis and interpretation of data. KM: acquisition of data. WB: conception and design, contributed unpublished essential data or reagents, analysis and interpretation of data, drafting or revising the article.

## Conflict of interest

The authors declare no conflicts of interest.

## Supporting information


**Figure S1.** Geographic and genetic distribution of 192 Arabidopsis accessions used in the study.
**Figure S2.** Experimental design of root phenotyping.
**Figure S3.** Effect of application of exogenous hormones on root architecture in 192 accessions, by root trait.
**Figure S4.** Variation of expression of key genes in the *SHY2* network in seven Arabidopsis accessions.
**Figure S5.** Sungear plot of all genes in the GWA regions of the four conditions.
**Figure S6.** Overlap of known ABA‐related genes and ABA GWAS candidate genes.
**Figure S7.** Effect of different MS concentrations on root growth.
**Figure S8.** Testing the effect of upper and lower row position in the plate.
**Figure S9.** One‐hundred and seven diverse Col‐0 trait values in comparison with those of other accessions.
**Figure S10.** Comparison of experimental reproducibility.
**Figure S11.** SNP patterns of ABA *bona fide* genes, all Arabidopsis genes and GWAS candidate genes.

**Table S1** List of 192 Arabidopsis accessions used in this study
**Table S2** Percentage of the reference accession Col‐0 within the total variation, by condition and root trait
**Table S3** Hierarchical clustering of Arabidopsis accessions by condition, with cluster numbers
**Table S4** PCA of root traits of 192 Arabidopsis accessions
**Table S5** Euclidian distances of treatment responses in the RSA space defined by PC1 and PC2
**Table S6** SNP/gene mapping for all significant associations
**Table S7** List of ABA *bona fide* genes
**Table S8** List of primer pairs used for qRT‐PCR
**Table S9** Root traits (mean) quantified in this study by accession/condition
