# Supplementary material for: Natural genetic variation shapes root system responses to phytohormones in Arabidopsis
Source: Plant J. 2018 Sep 10;96(2):468–81. doi: 10.1111/tpj.14034 (PMC6220887; doi:10.1111/tpj.14034)
Supplement: Supplementary file 1 — Figure S1. Geographic and genetic distribution of 192 Arabidopsis accessions used in the study. Figure S2. Experimental design of root phenotyping. Figure S3. Effect of application of exogenous hormones on root architecture in 192 accessions, by root trait. Figure S4. Variation of expression of key genes in the SHY2 network in seven Arabidopsis accessions. Figure S5. Sungear plot of all genes in the GWA regions of the four conditions. Figure S6. Overlap of known ABA‐related genes and ABA GWAS candidate genes. Figure S7. Effect of different MS concentrations on root growth. Figure S8. Testing the effect of upper and lower row position in the plate. Figure S9. One‐hundred and seven diverse Col‐0 trait values in comparison with those of other accessions. Figure S10. Comparison of experimental reproducibility. Figure S11. SNP patterns of ABA bona fide genes, all Arabidopsis genes and GWAS candidate genes. [file TPJ-96-468-s001.pdf]

A.

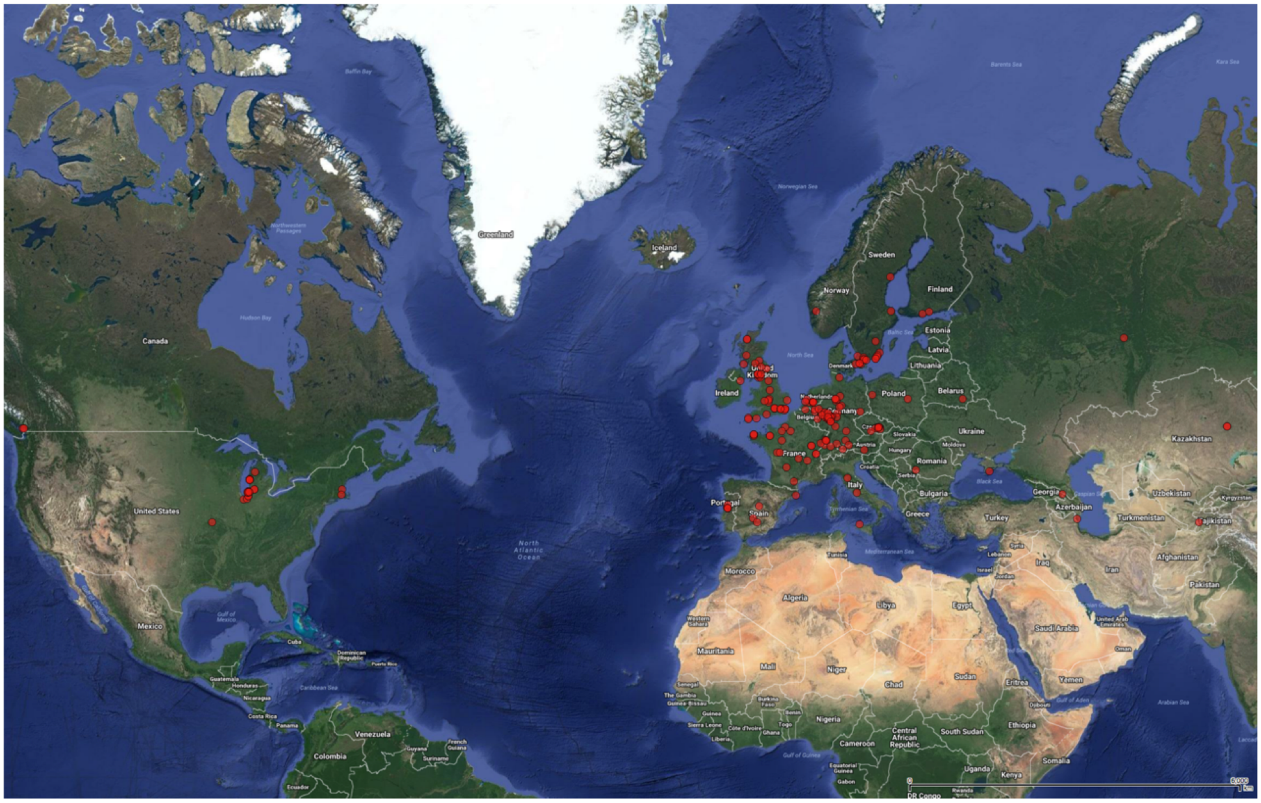

B.

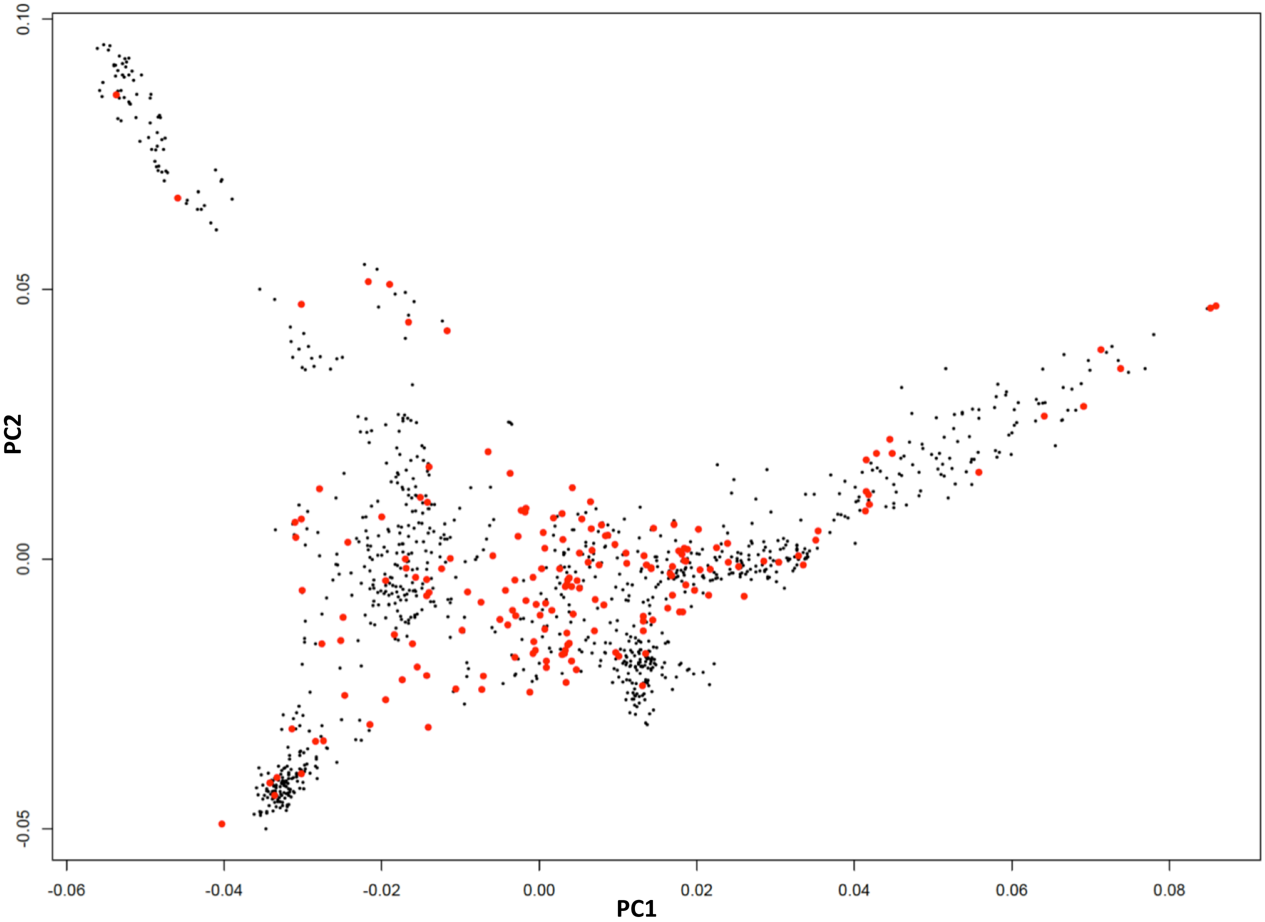

Supplemental Figure 1

A.

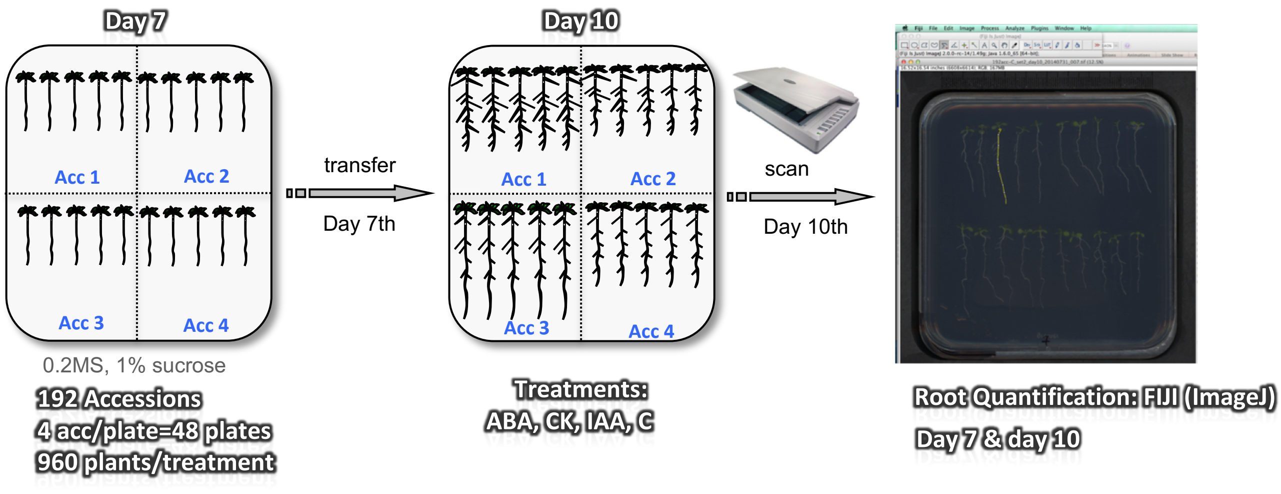

B.

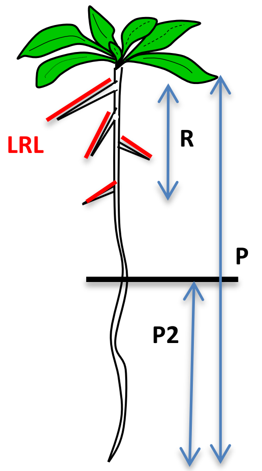

C.

Short Symbol of Trait	Name of Trait	Formula
P	Primary root length	-
P2	Growth of P after the transfer	-
R	Branching zone	-
LRL	Average lateral root length	-
LR.No	Lateral root numbers	-
TLRL	Total lateral root length	LR.L*LR.No
TRL	Total root length	TLRL+P
LRD_P	Root density in P	LR.No/P
LRD_R	Root density in R	LR.No/R
LRR	Length ratio	TLRL/P

Supplemental Figure 2

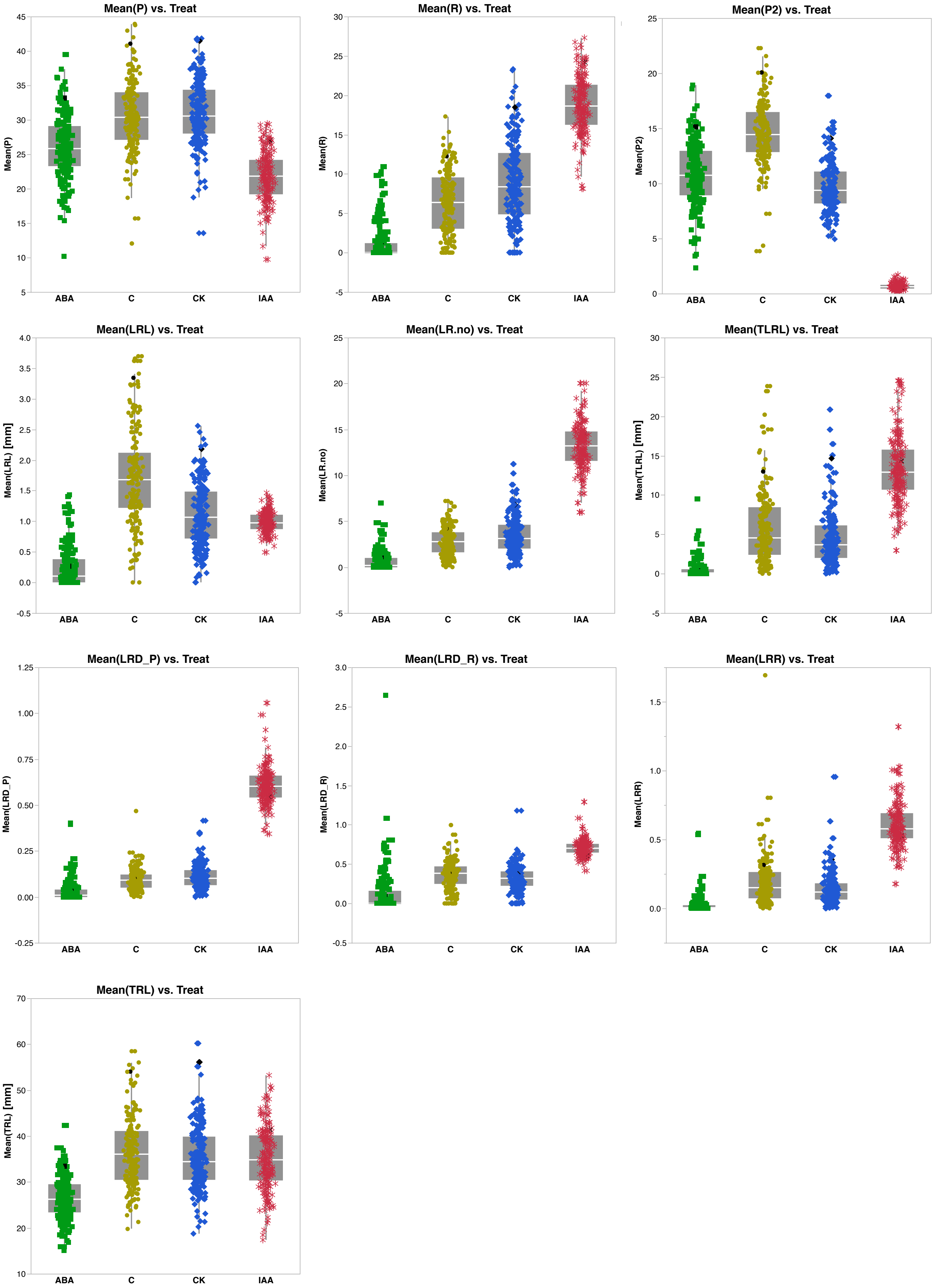

Supplemental Figure 3

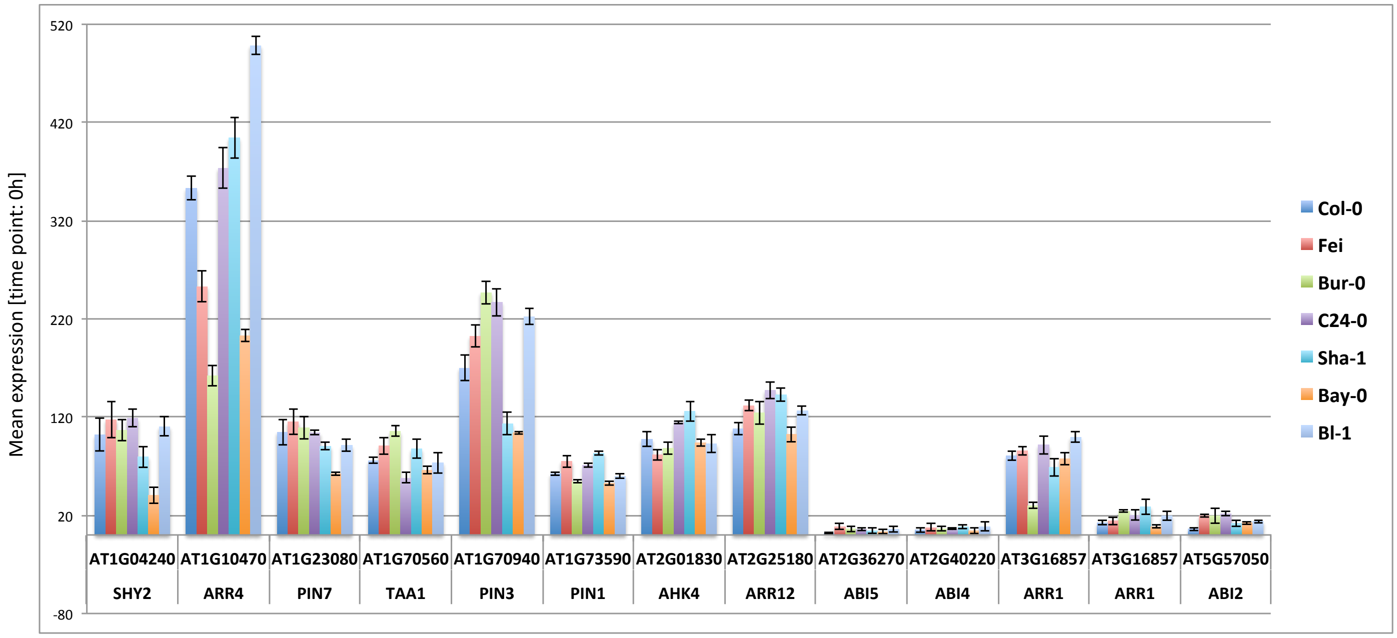

Supplemental Figure 4

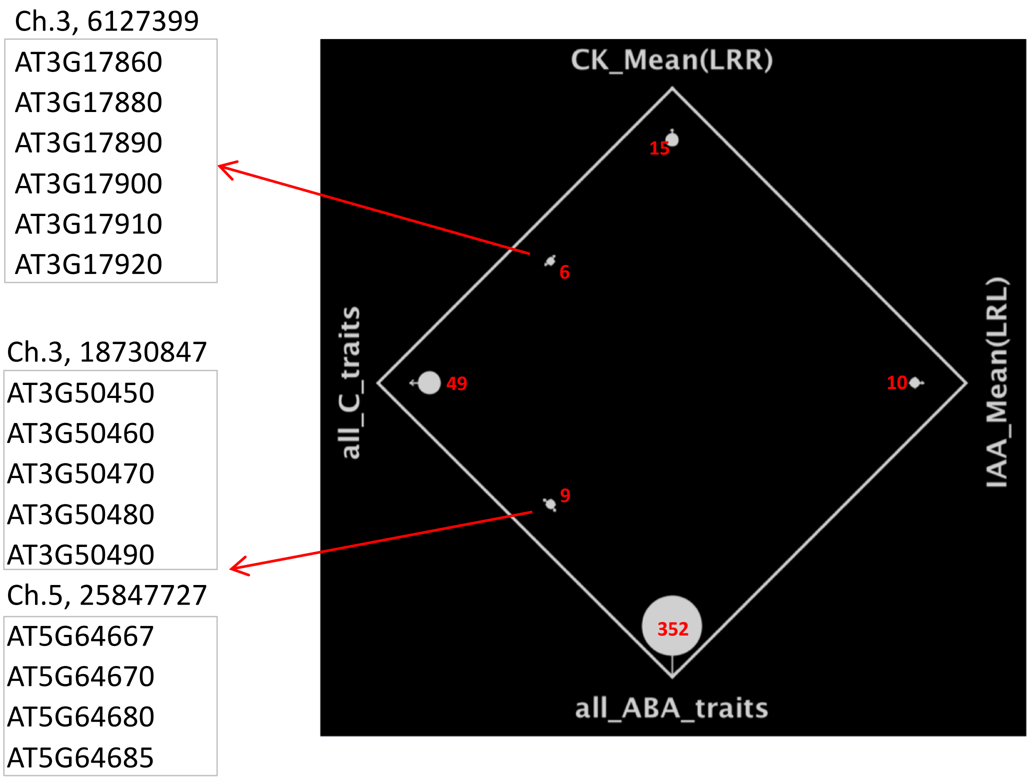

Supplemental Figure 5

A

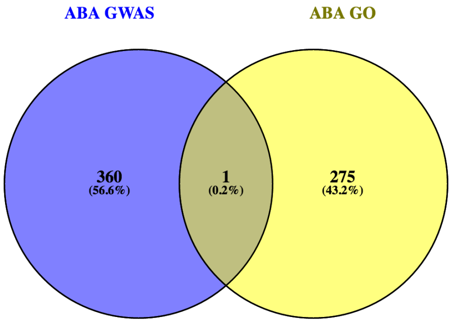

B

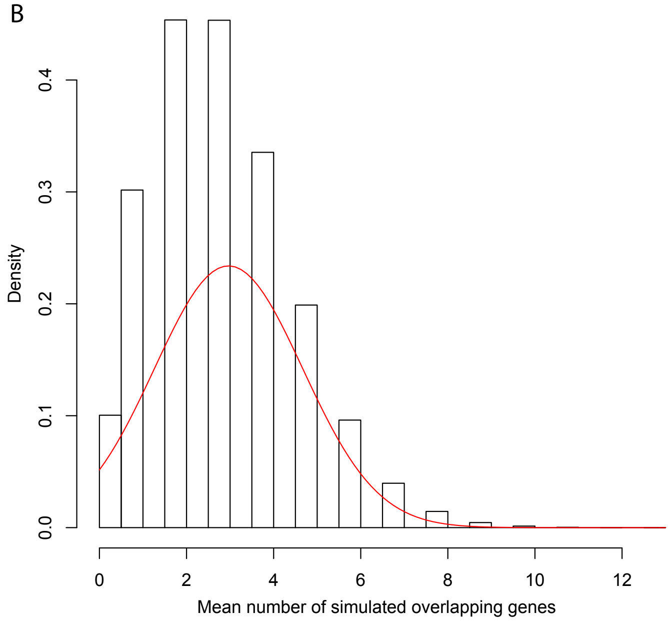

Supplemental Figure 6

A.

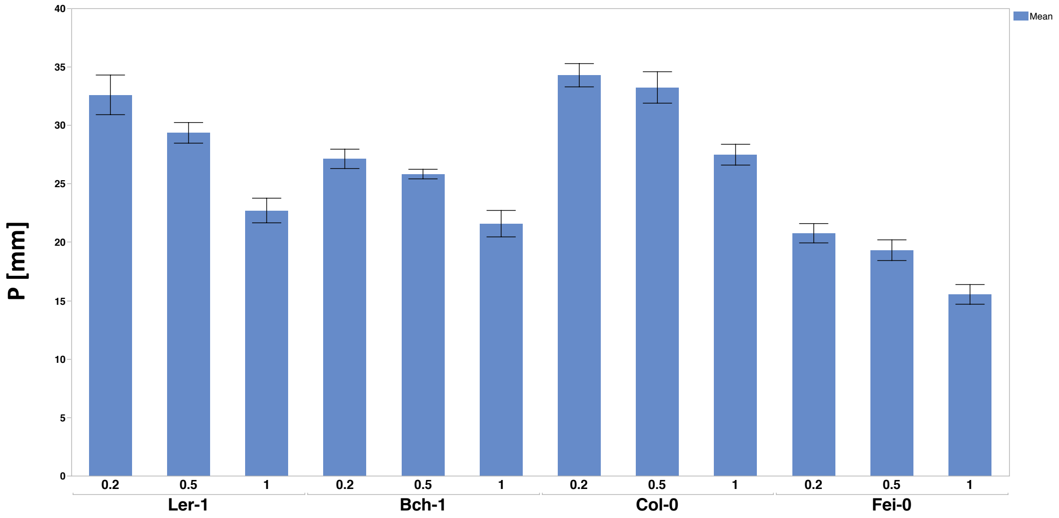

B.

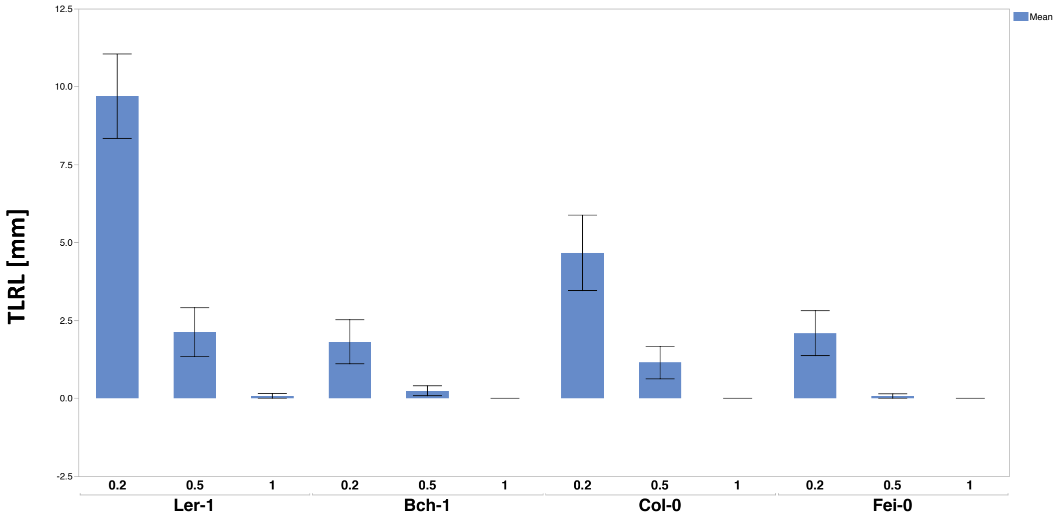

C.

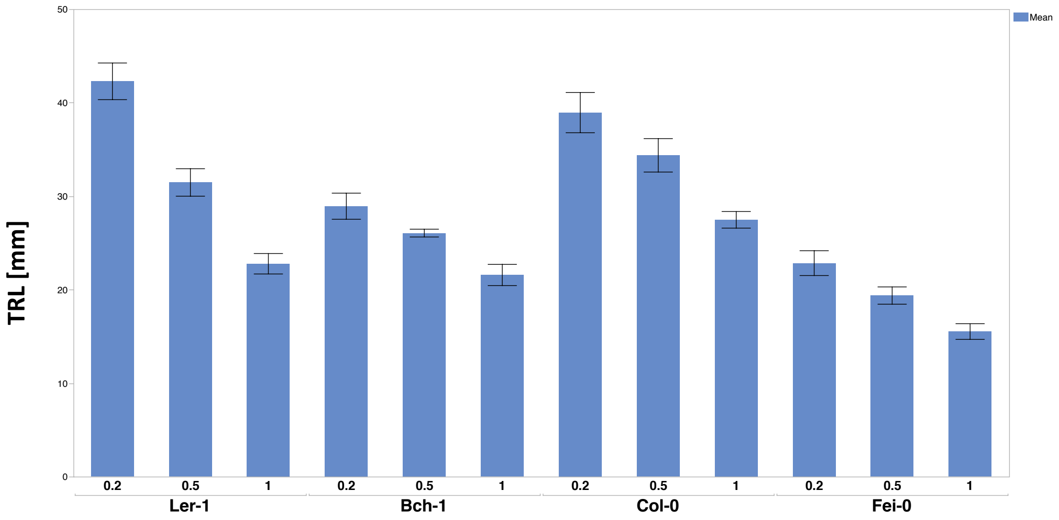

Supplemental Figure 7

A.

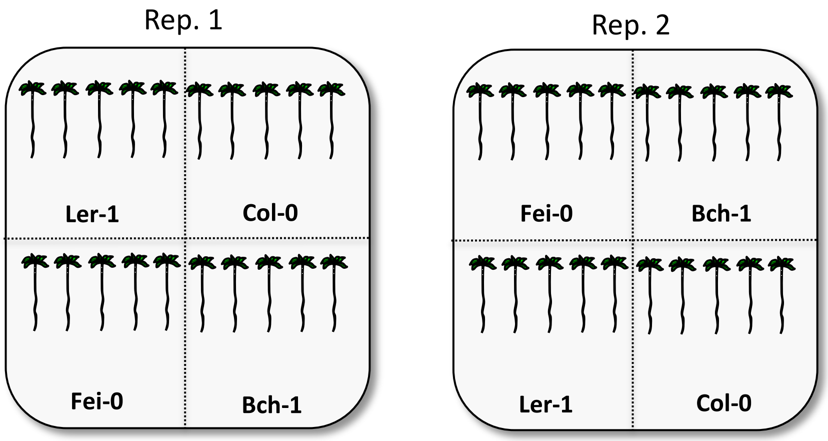

B.

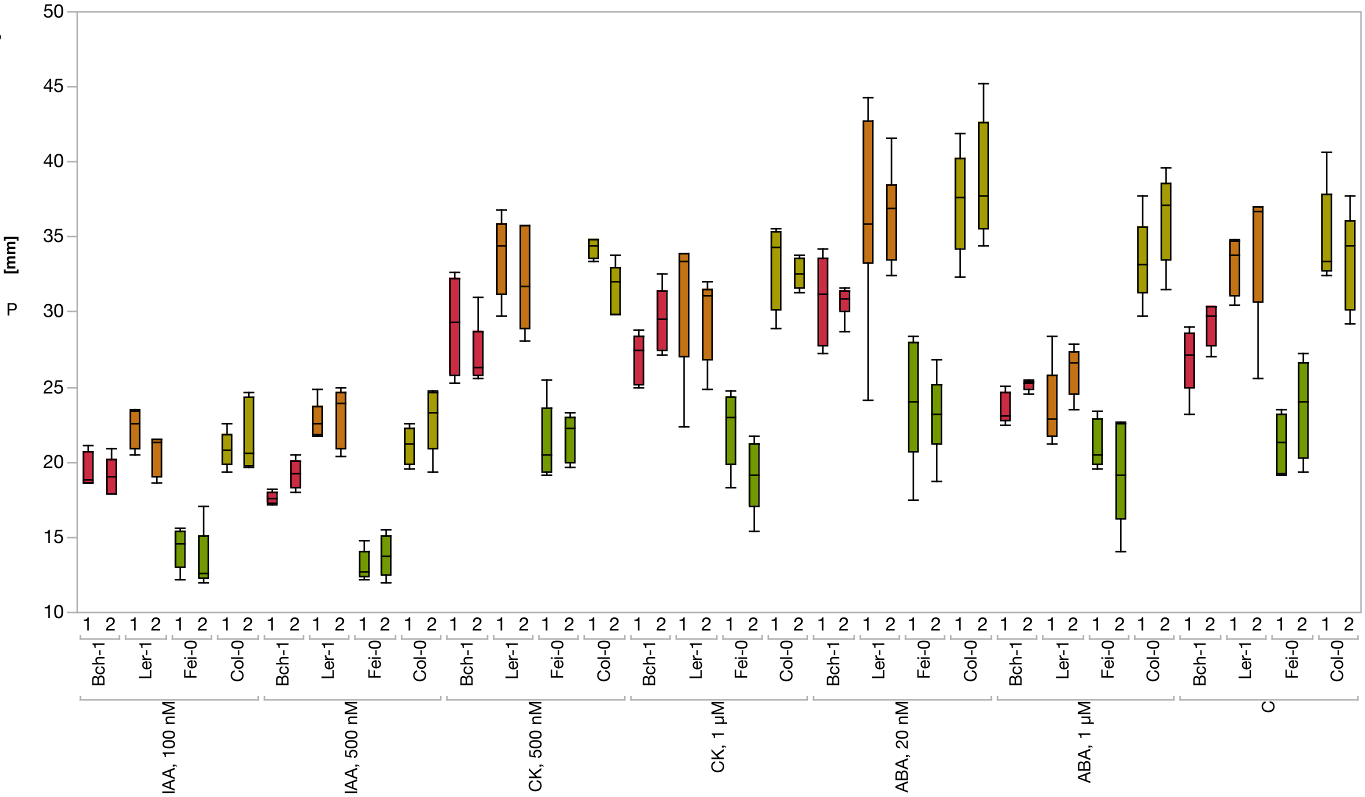

Supplemental Figure 8

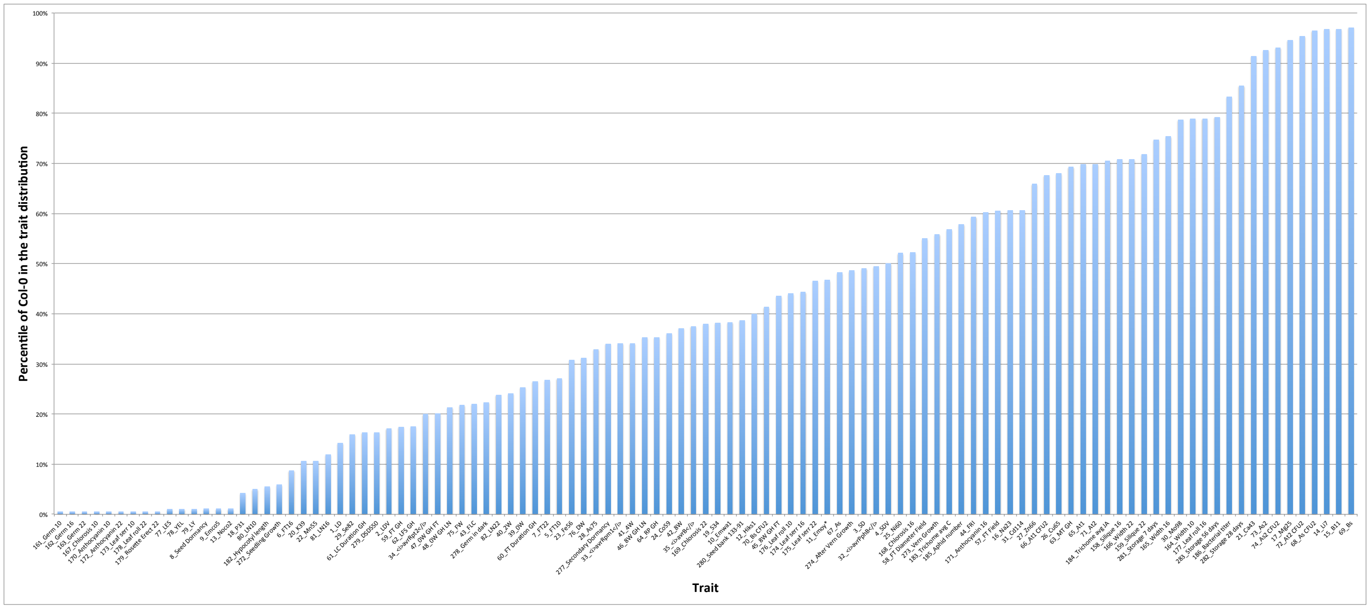

Supplemental Figure 9

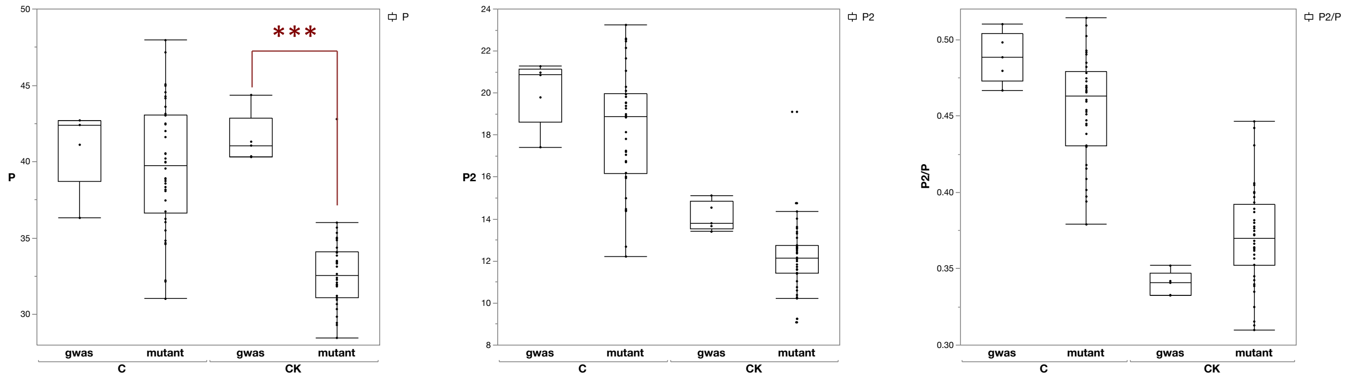

Supplemental Figure 10

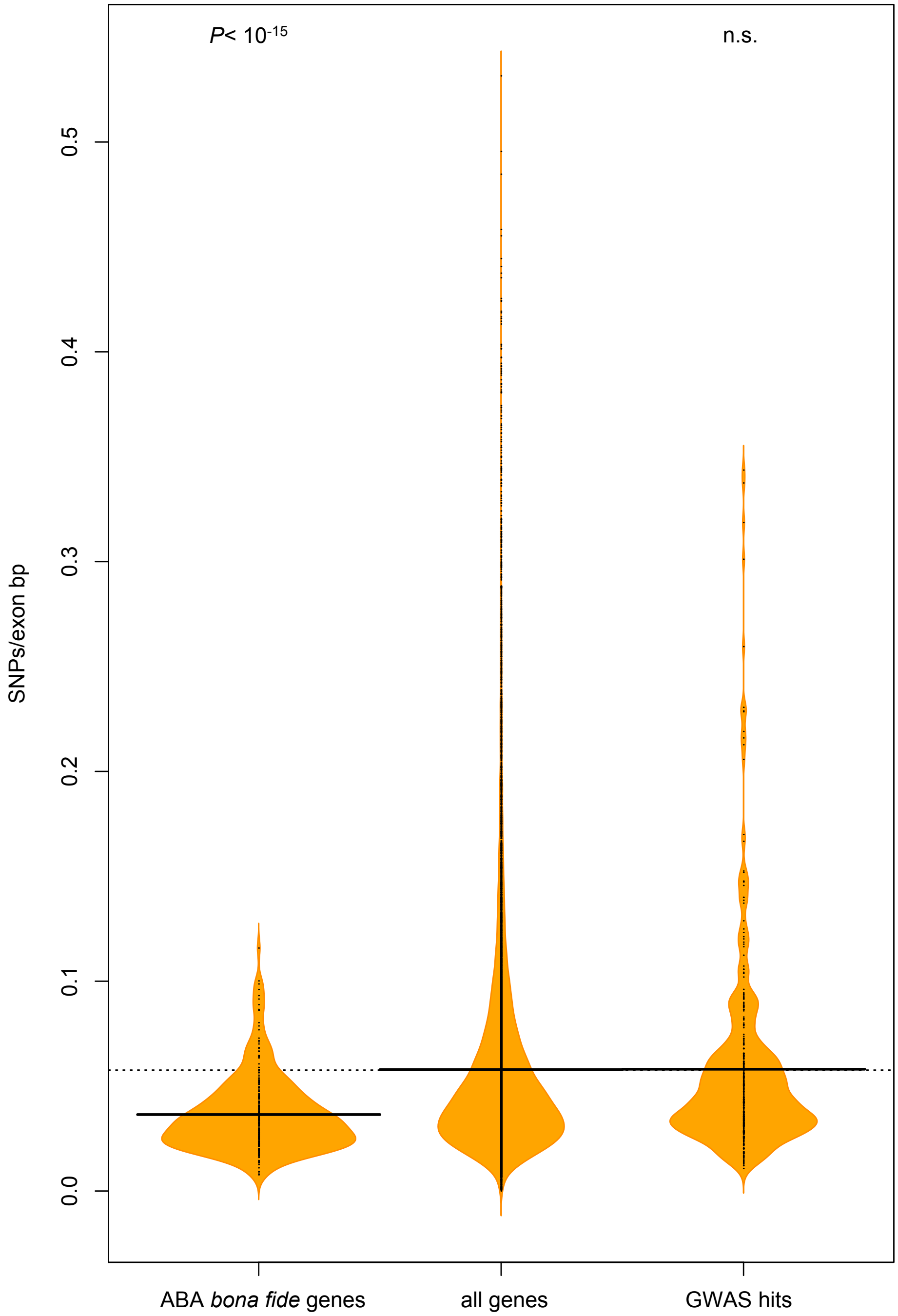

Supplemental Figure 11
